# Strengthening surveillance, disease detection, and outbreak response through Guinea-Bissau’s Frontline Field Epidemiology Training Program: a cross-sectional descriptive study

**DOI:** 10.11604/pamj.2023.45.133.30807

**Published:** 2023-07-19

**Authors:** Mamadú Camará, Fernanda Paulino da Costa, Geraldo Chambe, Agostinho Betunde, Placido Cardoso, Kenneth Johnson, Paola Rullan-Oliver, Augusto Lopez

**Affiliations:** 1 Guinea Bissau Frontline Field Epidemiology Training Program Frontline, National Institute of Health, Bissau, Guinea-Bissau,; 2 National Institute of Health (INASA), Bissau, Guinea-Bissau,; 3 Centers for Disease Control and Prevention, Atlanta, USA

**Keywords:** Guinea Bissau, FETP – Frontline, training, workforce development, field epidemiology, capacity building, public health, disease outbreaks

## Abstract

**Introduction:**

the goal of the Field Epidemiology Training Program (FETP) - Frontline is to strengthen the country's surveillance capacity at the district level to prepare and respond to health emergencies, including outbreaks, by training a skilled frontline public health workforce. We describe the FETP - Frontline program, including implementation, structure, achievements, impact, and its role in improving the epidemiological workforce capacity of Guinea-Bissau.

**Methods:**

this cross-sectional descriptive study uses 2015-2019 program data collected through record reviews and historical narratives from FETP students and graduates. We generated descriptive summary statistics using the Guinea-Bissau's FETP-Frontline program database, student assignments, and investigation reports, after reviewing the FETP standardized curriculum and program guidelines.

**Results:**

since its inception in 2016, FETP Frontline has implemented 14 cohorts and trained 198 frontline surveillance officers. Program participants improved surveillance data quality, investigated 51 outbreaks at national and regional levels, and contributed to disease research and surveillance in 227 separate field investigations. Participants frequently responded to priority health emergencies, including clusters or outbreaks of Zika, microencephalies, dengue, yellow fever, anthrax, malaria, and tuberculosis.

**Conclusion:**

Guinea-Bissau's FETP - Frontline program provides a practical example of an effective strategy to strengthen health systems through a well-prepared workforce trained to quickly detect and respond to health threats.

## Introduction

Guinea-Bissau is one of the smallest countries in West Africa, with an estimated population of 1.8 million inhabitants, divided into 8 administrative regions [1]. According to the World Bank, the country is classified as one of the poorest and most fragile countries in the world [2]. The country's epidemiological profile is characterized by a heavy burden of infectious diseases and a recent increase in non-communicable diseases. Life expectancy is estimated to be 61 years for women and 58 years for men. Neonatal mortality rates remain among the highest in the world, with an estimated 37 deaths per 1000 live births, and an <5 years mortality rate of 82 per 1000 live births [3]. The HIV burden in Guinea-Bissau is one of the highest in West Africa, with a 3.4% prevalence among adults aged between 15 and 49 years [4,5]. The inability of the health system to provide an adequate response leads to high morbidity and mortality rates, particularly from acute respiratory infections (ARI), malaria, and neonatal conditions. Other factors that underlie the country's high disease burden include environmental conditions, alcohol and drug consumption, and an unbalanced diet.

The national health system comprises three levels of care: central, intermediate, and peripheral. At the *central level*, there is the Ministry of Public Health (MINSAP) and the National Institute of Public Health (INASA). The INASA was established in 2011 as a government agency that has authority over epidemiology, surveillance, health communication, public health laboratory system, and health research. The *intermediate level* consists of 11 health regions, including the Autonomous Sector of Bissau (SAB). Health regions offer technical support and coordination of health areas that constitute the *peripheral level* [6]. Each of the 114 health areas has at least one health center. Regional, rural, and urban differences exist in the health indicators of the population, the existing care delivery infrastructure and respective functioning, and the resources available for each setting. One of the main challenges at the peripheral level is the weak health information system due to an untrained workforce, lack of reporting, poor data quality, and other factors.

Guinea-Bissau uses the Integrated Disease Surveillance and Response (IDSR) strategy to detect priority diseases, conditions and events, and report across all health system levels in the country [7]. This includes cholera, dysentery, measles, pertussis, meningitis, acute flaccid paralysis/polio, neonatal tetanus, non-seasonal flu, hemorrhagic fever, rabies, acute diarrhea, microcephaly/Zika, and maternal death [8]. The 2014 Ebola outbreak demonstrated the need for improved field epidemiology capacity at all levels of the health system in the West African region. There was a shortage of appropriately trained staff and a lack of timely disease detection and response [9,10]. In addition, Guinea-Bissau shared a border with affected countries, namely Guinea-Conakry and Senegal. Therefore, the World Health Organization (WHO) declared Guinea-Bissau as one of the countries in Africa with a high risk of spreading Ebola [11,12].

In January 2015, the US Centers for Disease Control and Prevention (CDC) and the Global Network of Training Programs in Field Epidemiology and Public Health Interventions (TEPHINET) implemented an emergency training strategy - Surveillance Training for Ebola Preparedness (STEP) - to rapidly increase surveillance capacity in health regions and along areas bordering affected countries [9,10]. The STEP program focused on training epidemiology personnel at the district and regional levels, who were usually the focal points for compiling, analyzing, and reporting disease surveillance and outbreak data [9]. In 2016, based on the experience of the STEP training and with technical support from CDC, Guinea-Bissau's Ministry of Health (MINSAP) officially established the Field Epidemiology Training Program-Frontline level (FETP-Frontline) in the country. FETP-Frontline is recognized worldwide as an effective strategy to strengthen a country's public health surveillance system in a short time. It has a demonstrated impact on the number of qualified health professionals able to effectively and rapidly detect and respond to public health emergencies [9]. The program was implemented with a One Health perspective, training experienced professionals from multiple health sectors, including human health, animal health, laboratory, and social sciences [11].

This paper aims to describe the implementation of FETP-Frontline in Guinea-Bissau, its development and organization, the major outputs of the program, and its contribution to the country's public health system. Our main hypothesis was that FETP-Frontline will contribute to a stronger public health system through improved disease surveillance, data collection, analysis, reporting, and frontline response.

## Methods

This descriptive study documents the history and functionality of Guinea-Bissau's FETP-Frontline between 2015 and 2019. For secondary data analysis, we accessed the program's student and alumni database, program guidelines, classroom and field assignments, and investigation reports completed during the implementation of the program. We used to Microsoft Excel for data management and to perform summary statistics of participant profiles between 2016-2019, including the number of cohorts, graduates by profession, and activities performed. We used QGIS^2^ to visualize the geographic distribution of trained FETP-Frontline graduates. We also reviewed the program guidelines to describe the planning, curriculum, and implementation process. FETP-Frontline participants were selected based on recommendations from INASA and other health institutions, availability, interest, and minimum skillset to complete the program.

**Funding:** the funding for the Guinea-Bissau FETP was provided by the United States Centers for Disease Control and Prevention (US CDC) through a cooperative agreement with AFENET number CDC-RFA-GH15-1619 for Strengthening Applied Epidemiology and Sustainable International Public Health Capacity through Field Epidemiology Training Programs.

**Disclaimer:** the findings and conclusions in this manuscript are those of the author(s) and do not necessarily reflect the official position of the Centers for Disease Control and Prevention.

## Results

### Program description

***Field Epidemiology Training Programs (FETP)*** *are 3-tiered in-service training programs for public health professionals to strengthen their field epidemiology and surveillance skills. The goal of FETP is to build field epidemiology capacity at all levels of a country's health system, starting from the district (FETP-Frontline) to the regional (FETP-Intermediate) and the national (FETP-Advanced) levels*.The FETP-Frontline in Guinea-Bissau aimed to develop and strengthen the epidemiological capacity in the country to strengthen public health surveillance and evidence-based decision-making, particularly at the local level of the surveillance system. The program is coordinated by the INASA and receives technical and administrative assistance from CDC-Atlanta and the African Field Epidemiology Network (AFENET), respectively.

***Program selection:*** the FETP-Frontline integrated training for human and animal health professionals to enhance global health security from different and complementary viewpoints [8,11]. The target audience included public health professionals at the central and regional levels who are responsible for compiling and analyzing surveillance data, and local public health professionals responsible for surveillance, data collection, compilation, analysis, reporting, and response at the local level of the health system. INASA and other health institutions or regions recommended candidates for consideration into the program. Based on the indication, the FETP program team evaluated the candidates to assess availability, interest, and minimum skillset to complete the course.

***Guinea-Bissau's FETP-Frontline Program:*** Guinea-Bissau's FETP-Frontline used the CDC standardized curriculum translated to Portuguese and adapted to Guinea-Bissau's training needs [13]. The three-month long service training followed the “learning-by-doing approach,” where up to 80% of the time is spent on practical activities and field projects.

The **standardized curriculum for FETP-Frontline** is divided into 3 classroom workshops and 2 fieldwork blocks in between. The program provided additional workshops in GIS and Epi-Info. During the training process, the participants learned and practiced fundamental epidemiological skills used in surveillance including disease detection, reporting, and case investigation and response at the local level. They used case definitions, simple tables, and graphs to apply their skills to summarize and present epidemiological data.

**Workshop 1** was held in 5 days, where we covered surveillance data collection, analysis, and interpretation, data quality, and basic principles of biostatistics. After this workshop, the professionals returned to their field of work, usually related to epidemiological surveillance. During the 4 weeks of fieldwork, the students conducted a data quality audit and a surveillance data analysis summary report.

**Workshop 2** also lasted 5 days. It was an opportunity for participants to present their data analysis and delve deeper into surveillance questions and discussions. They were also introduced to the basic concepts of scientific communication and case/outbreak investigation and response. After the second workshop, residents returned to their workplace, where they conducted field projects to practice, implement, and reinforce what they learned. This included participating in outbreak investigations and/or conducting case investigations, while ensuring thorough analysis of surveillance data.

The course finalized at **workshop 3**, where the professionals presented their results to FETP colleagues, country's health managers, stakeholders, and local health authorities. Graduating participants received a course completion certificate. At the end of the program, each participant submitted the following products: (1) Data quality evaluation report with recommendations for improvement; (2) Surveillance summary report; (3) Outbreak investigation report; (4) Oral presentation of the surveillance summary.

The classroom sessions were conducted by epidemiologists and public health professionals from different health agencies, such as Guinea-Bissau's FETP-Frontline graduates, CDC Atlanta, World Health Organization (WHO), INASA, National Public Health Laboratory, Portuguese Cooperation, Bandim Health Project, and Médecins Sans Frontière (MSF). During the program, each participant was assigned a mentor who was in regular contact with them and provided feedback and guidance, as needed, for the successful completion of the field project. The ratio of mentors to mentees was 1: 3.

**Pre and post-test questionnaires**, completed by trainees at the beginning of the workshops and upon return from the field, were used to evaluate the course. Evaluation questions were related to the content covered in each of the workshops and the practical activities. Results from the questionnaires were presented during the final workshop, where residents had an opportunity to discuss and request clarifications. For each cohort, we completed a post-training evaluation to assess the impact of the course on the graduates' work routine. A strategy is being developed for this type of evaluation to be incorporated into the standardized program curriculum.

**Figure 1:**
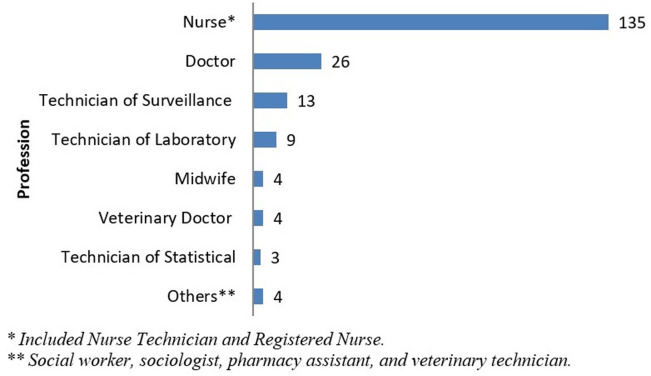
profession of graduates of FETP-frontline Guinea-Bissau, 2016 to 2019

***FETP-Frontline implementation:*** in November 2016, CDC completed an in-country assessment to implement the FETP program in INASA. In addition, a resident advisor was hired to support program implementation. In March 2016, FETP-Frontline was officially launched. In 2017, the program expanded, adding 2 regional and 2 national cohorts. From 2016 to 2019, Guinea-Bissau's FETP-Frontline implemented 14 cohorts and successfully trained 198 graduates. The program trained professionals from the national or regional levels (6 cohorts) and from the local level (8 cohorts) of the health system. All graduates conducted a descriptive study with surveillance data and 83 (42%) participated in at least one outbreak investigation or emergency response in public health (Table 1). Regarding professions, 81% (161/198) of the graduates were either nurses or physicians (Figure 1). The trainees completed the fieldwork in their respective workplaces, which included MINSAP, INASA, health regions, local health areas, hospitals, military health, and animal health (central and regional levels of the Ministry of Agriculture). There were also several challenges associated with the implementation of Guinea-Bissau's FETP-Frontline. Ensuring long-term program sustainability was the main one. The implementation process highlighted the limited diagnostic capacity of laboratories; lack of qualified, committed, and available mentors; limited human resources; and difficulty publishing study findings in indexed international journals given that the FETP-Frontline residents are non-native English speakers.

**Table 1:** number of cohorts, graduates, and activities developed from 2016 to 2019, FETP-frontline, Guinea-Bissau

Year	Nº cohorts	Nº graduates	Nº activities	
			Descriptive study with surveillance data	Outbreak investigation or emergency response
**2016**	3	39	39	19
**2017**	4	62	62	24
**2018**	6	87	87	37
**2019**	1	10	10	03
**Total**	**14**	**198**	**198 (100%)**	**83 (42%)**

***Guinea-Bissau's FETP-Frontline main outputs:*** student projects and fieldwork covered reportable infectious diseases, such as malaria, Zika, dengue, chikungunya, yellow fever, tuberculosis, HIV/AIDS, leprosy, leishmaniasis, and measles. Other health topics included carbuncle, noma, diabetes, hypertension, maternal health, general mortality, human anger, and border surveillance. In 14 cohorts, Frontline trainees completed a total of 227 field investigations, including 51 outbreak investigations, while responding to public health emergencies (Table 2). Since the beginning of the program, all outbreaks detected and reported to INASA have been investigated with the support of FETP-Frontline residents. Residents investigated outbreaks including measles, cutaneous anthrax, foot-and-mouth disease (FMD), rabies, various food intoxications. This has also created an opportunity for One Health coordination and collaboration between the human and animal health sectors (Table 3).

**Table 2:** number of Field investigations conducted by 14 cohorts of FETP-frontline in Guinea Bissau, 2016-2019

Field Investigations	No	%
Communicable Diseases	83	37
Maternal-Child Health	55	24
Outbreak Investigations	51	23
Public Health	15	7
Non-Communicable Diseases	11	5
Laboratory	7	3
Animal Health	5	2
**Total**	**227**	**100**

Source: Database of graduates of FETP-Frontline. Guinea Bissau.

**Table 3:** outbreak investigations conducted by FETP-frontline graduates, 2016-2019, Guinea-Bissau

Outbreak	No	%
Vector-borne diseases	27	53
Vaccine preventable diseases	11	22
Foodborne diseases	8	16
Zoonotic diseases	5	10
**Total**	**51**	**100**

Source: Database of graduates of FETP-Frontline. Guinea Bissau.

**Figure 2:**
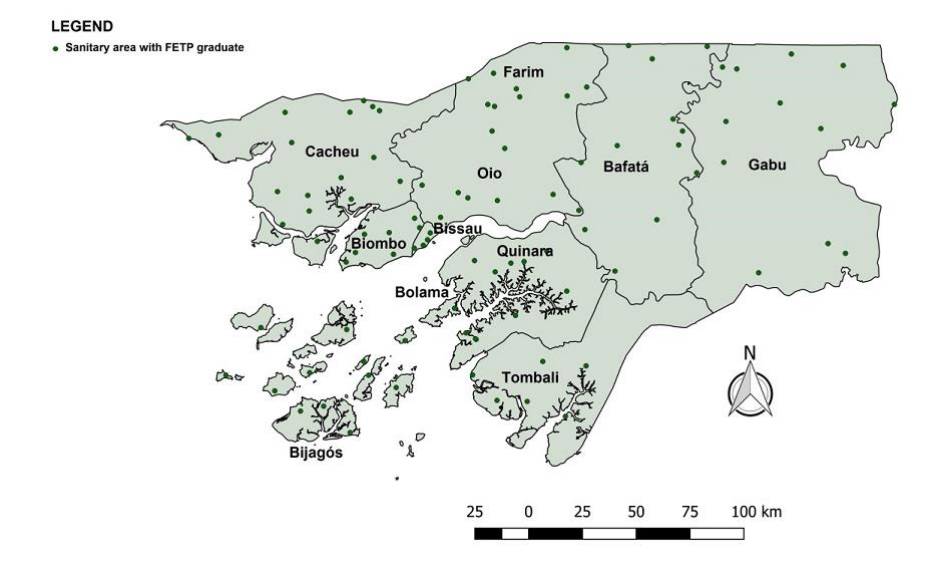
Guinea-Bissau's health areas with at least one FETP-Frontline graduate, 2016-2019 (10 areas are not georeferenced)

In 2016, the rapid international spread of Zika highlighted the need for rapid detection and monitoring of infectious diseases. The FETP-Frontline confirmed the first cases of Zika in the country, demonstrating the importance of a highly trained, robust public health workforce of disease detectives working to rapidly identify, track, and limit disease spread [14]. Recommendations from the fieldwork were shared with stakeholders and other decision makers at various forums, including workshops and dissemination meetings. From 2016-2019, 193 human health professionals and 5 animal health professionals graduated from the program and currently hold key surveillance and emergency response positions across the country. Each of the 38 districts and 8 health regions has at least 2 graduates (1 in STEP and 1 in FETP) from the program who collect and analyze epidemiological data (Figure 2). They also participate in outbreak investigations, improving the country's capacity to prepare and respond to epidemiological situations.

***International Collaboration and Networking:*** in May 2017, we established a collaboration plan between Guinea-Bissau's FETP-Frontline and Brazil's FETP (EpiSUS) within the framework of the Lusophone FETP Network. The main objective is to strengthen Guinea-Bissau's FETP and the overall public health system, by training Guinean professionals in Brazil's FETP Advanced 2-year program. The first medical professional who completed the course in Brazil returned to Guinea-Bissau, where he assumes a surveillance management post. In 2020, two additional professionals completed Brazil's FETP Advanced training, and three other Guinean residents graduated from the West Africa Regional Advanced FETP Program, located in Burkina Faso. Guinea-Bissau's FETP-Frontline has been an AFENET and TEPHINET member since 2017, where they can participate in conferences, receive technical support and funding, and network with other programs.

## Discussion

In 2016, the FETP-Frontline in-service training strategy was successfully implemented in Guinea-Bissau and supported the country's effort to respond swiftly to priority public health events. This paper describes the implementation of the program, its development, organization, main outputs, and its contribution to the country's public health system from 2016-2019. During this time period, the program successfully trained 198 graduates in FETP-Frontline where each participant conducted a descriptive surveillance project and 42% assisted in outbreak investigations or emergency public health responses. Globally, FETP-Frontline has proven to be a practical solution to improve disease surveillance and response in low- and middle-income countries [9,15]. Guinea-Bissau's high-level government recognition and the international support from TEPHINET, AFENET, CDC, WHO, and other partners, emphasize the program's value and impact.

The main challenge with implementing Guinea-Bissau's FETP-Frontline is guaranteeing the program's sustainability. The program receives financial support, transited from CDC funds to the World Bank, through the Regional Disease Surveillance Systems Enhancement (REDISSE). However, the goal is to build public health capacities independent to foreign aid and support. The country may consider how the FETP can be incorporated and sustained within the country's current health system. Therefore, a steering committee was established to discuss how to strengthen the program and establish it into the national surveillance network.

Second, Guinea-Bissau has limited laboratory diagnostic capacity, which the government is working hard to improve. Some disease samples must be sent to the Institute Pasteur in Dakar, Senegal, for diagnostic confirmation. Laboratory diagnosis is a key component in outbreak investigations, and delays in receiving results from reference laboratories limits the interpretation of epidemiological data.

Third, few graduates have the availability, financial resources, and adequate technical preparation to become mentors and continue to support the program and One Health approach to field epidemiology. Out of the 198 Frontline graduates, only 9 worked in laboratory settings, 5 worked in the animal health sector, 2 in the social sciences. Identifying and maintaining highly qualified and committed mentors is still a major concern among FETPs globally [9,16,17]. Similarly, this has been seen in many newly formed programs, mainly in the African context. Mentorship needs to be strengthened and valued as a key part of the program's success, through regular program evaluations and incorporation into the health system's long-term sustainability plan. International and cross-sectoral collaboration for advanced level training increases the number of professionals able to develop mentoring activities in the country and across different health sectors.

Fourth, the technical and operational capacity of human resources is limited at the national and sub-national levels. Retaining graduates within the health system is key for the program's long-term objectives of strengthening the health system, including successful mentorship.

Finally, one of the principles of epidemiology in public health is information sharing of relevant study findings through reports, publications, and other means of scientific communication. Given that trainees of Guinea-Bissau's FETP-Frontline program are not native English speakers, they face challenges in publishing their field work with the broader public health community. To overcome this obstacle, a Scientific Writing Program and Calibrated Peer Review exercises, can help improve scientific writing skills for residents to showcase their research in the international arena [18,19].

The national limitations of the capacity to respond to outbreaks, perform laboratory investigations, and implement fieldwork recommendations have prevented the progress of some studies. However, the program's benefits are unquestionable, as they strengthen and build epidemiological capacity and public health surveillance. Guinea-Bissau's pyramid model of field epidemiology training (Frontline, Intermediate, and Advanced) is still underdeveloped, but is expected to implement the next tier, FETP Intermediate, by 2024.

## Conclusion

FETP-Frontline in Guinea-Bissau is an effective strategy to respond to public health emergencies and improve a country's local surveillance system, through in-service training of health professionals involved in data generation, analysis, scientific communication, and evidence-based recommendations for decision making. However, to sustain the FETP-Frontline program in the long term, it is essential for the country to have a structured programmatic approach that considers the program's financial, operational, and technical sustainability.

### What is known about this topic

- *The purpose of FETP-Frontline is to strengthen a country's surveillance system at the local level;*
- *Committed and experienced mentors, as well as selecting suitable trainees, are important to ensure the quality and impact of the program*.

### What this study adds

- *Guinea-Bissau's FETP-Frontline experience confirms the importance of strengthening surveillance at the local level;*
- *The FETP-Frontline training course has improved the country's ability to rapidly detect and respond to health emergencies in Guinea-Bissau*.
